# Book Reviews

**Published:** 1828-07

**Authors:** 


### CRITICAL ANALYSES.

Quæ laudanda forent, et quæ culpanda, vicissim

Illa, prius, cretâ; mox hæc, carbone, *notamus*.—Persius.

**_Surgical Observations on the Treatment of Chronic Inflammation in various Structures; particularly as exemplified in the Diseases of the Joints._ By John Scott, Surgeon to the London Ophthalmic Infirmary; and Assistant Surgeon to the London Hospital.—8vo. pp. 291. Longman and Co. London, 1828.**

For a very considerable period, Mr. Scott, of Bromley, has enjoyed extensive reputation for his successful treatment of various diseases, but particularly of different affections of the joints. His plan of treatment, however, has not been distinctly known to the great body of the profession. From persons who have been under his care, we have often heard the most contradictory accounts both of his principles and practice; while, with very few exceptions, they have all agreed that they have derived much benefit at his hands, even when the first chirurgical talent of the metropolis had been in vain exerted to relieve them.

We are too much accustomed to the exaggerated reports of patients, to rely implicitly upon them. It would be idle, indeed, to expect correct information upon such subjects from public rumor. As the work before us comes from the pen of Mr. Scott's son, we shall no longer be in doubt concerning a mode of treatment, about which so much has been said, but of which so little has been positively known.

The author compliments Mr. Brodie for his accurate investigation of the pathology of diseases of the joints, but he conceives that the treatment still admits of great improvement. He observes, that the most profound pathologists are not always the most successful practitioners; and in the truth of this sentiment we cannot but agree, without, however, the most distant intention of any individual application of it. The fact has been impressed upon us by frequent observation.

The object of the present work must be anticipated by our readers. It is to communicate the mode of treatment Mr. Scott, of Bromley, has for many years employed in diseases of the joints, with complete success in a vast number of cases "in which the methods ordinarily employed had proved ineffectual." The author has seen its efficacy in many cases of his father's practice, and subsequently in his own, and "he feels it to be too important to be confined to an individual." After such an exordium, we naturally give our attention to the work with additional curiosity, and with increased expectation of the value of the information we shall be enabled to convey to our readers.

It has been deemed necessary to prefix a brief inquiry into the nature and treatment of chronic inflammation, and ulceration in general, in order that the operation of the remedies proposed may be satisfactorily explained. We are informed that "the influence of disorder of the health and the digestive organs in keeping up local diseases, has of late years been fully explained; but little notice has been taken of the reverse truth, the influence of local disease in keeping up disorder in the constitution and the digestive organs." It may be true that no work has been particularly dedicated to the consideration of the latter fact, but we cannot admit the inference that it does not exert its due influence with every attentive, well informed, and unprejudiced practitioner. It is an admitted axiom, although volumes may not have been expended in support of it, that the stomach has as much to apprehend from local disease, as local disease from the stomach.

"Pain or irritation in any part will assuredly spread disturbance throughout the system, and thereby impair the functions of the stomach, and its connected organs; and if we can relieve this pain, or soothe this irritation, by local remedies, we shall go as far towards imparting tranquillity to the system and the stomach as by the employment of alteratives, aperients, and a regulated diet, which it is often vain to adopt without attention to the former. There never was a greater delusion than that of supposing, with some modern surgeons, that medicine and diet are all that is necessary for the treatment of local diseases, and that local remedies are needless. It is a scrupulous attention to, and a dexterous application of, the latter in addition to the former, which has enabled my father to succeed in curing so many local diseases, which had baffled all previous efforts." (*Preface*, p. viii.)

A neglect of local remedies, especially during the existence of much local irritation, is a practical error, which they only can commit who believe, or at least assert they believe, that the sole business of the medical or surgical practitioner is to regulate the condition of the stomach and bowels. Such partial views have, however, but few advocates, and we are much mistaken if their opportunities of practically enforcing them have not been lately on the decline.

We must be brief in our notice of the introductory observations upon chronic inflammation, and its treatment.

Chronic ulceration of the lower extremities frequently bids defiance to the ordinary treatment, and no doubt cases of this sort are more usually treated empirically, than upon well understood pathological principles. Mr. Scott considers the ulcer to be "only the termination and effect of the chronic inflammation by which it is surrounded, and the former cannot be healed until the latter is removed. In the treatment, the direct object is not to heal the ulcer, but to cure the chronic inflammation; for, if this can be effected, the ulcer heals spontaneously. The essential remedy for this state of things is mechanical support, which restores to the vessels the power of propelling their fluid along their canals." (P. 5.) It is well known that for the general adoption of this mode of treatment we are indebted to the late Mr. Baynton, of Bristol. Mr. Scott is sensible of the practical advantage of the remedy recommended by Mr. Baynton, but he differs from him as to the mode of its operation. He concludes that the effusion of lymph and of serum into the cellular membrane, and the distention of the integuments, are the effect, not the cause, of inflammation, as Mr. B. supposed. Mechanical support is equally well adapted to ulcers on the lower extremities, whether they arise from a varicose state of the veins or not. In many cases of chronic inflammation not so violent as to produce ulceration, it will also afford great relief.

"In the former cases, it is not the ulceration that is the object of our solicitude, but the inflammatory action which induces ulceration. The ulceration ceases as soon as the inflammation is arrested; and, as this has been shown to depend on distention of the views, which are no longer able to resist the gravitation of the blood, we have only to afford such an uniform support to the limb as shall prevent the veins from yielding to the pressure of their contents. If we adopt the adhesive bandage with this view, it must be applied in a manner very different from that in which it is recommended by Mr. Baynton. He directs the 'middle of the piece of plaster to be applied to the sound part of the limb, opposite to the inferior part of the ulcer, so that the lower edge of the plaster may be placed about an inch below the lower edge of the sore, and the ends drawn over the ulcer with as much gradual extension as the patient can well bear. Other slips are to be secured in the same way, each above and in contact with the other, until the whole surface of the sore and the limb are completely covered, at least one inch below, and two or three above, the diseased part.'—'The force with which the ends are drawn over the limb must be gradually increased, and, when the parts are restored to their natural ease and sensibility, which will soon happen, as much may be applied as the calico will bear, or the surgeon can exert.'" (P. 9.)

Mr. Scott deprecates this mode of applying the plaster bandage. He has seen instances in which great mischief has arisen from it.

"The pressure round the part of the leg encircled by the plaster and bandage is so much greater than at the lower part, where a roller only is applied, that the venous circulation is so much impeded as to cause considerable tumefaction of the foot and ankle. This produces extensive inflammation, which is propagated to the original seat of disease. Besides, in many instances, the inflammation of an ulcerated leg extends much more than an inch below the ulcer; so that, according to Mr. Baynton's directions, we are to apply to a portion only of the disease a remedy which, when so applied, aggravates the remainder; for I repeat that inflammation is the disease, and ulceration only its consequence." (P. 11.)

Instead of commencing the application of plasters an inch below the ulcer, it is necessary, according to Mr. Scott's view, to afford equal support to the whole limb, in order effectually to bring about a uniform state of the circulation.

"With regard to the method of fulfilling the foregoing indication, the Emplastrum Plumbi, P. L., spread on calico, is the best application, as it does not irritate the skin. It is most conveniently made use of when cut into slips of fifteen inches in length, by two in breadth. The foot being placed at a right angle to the leg, one of the slips should be applied from the first bone of the great toe, along the inner edge of the foot, around the posterior part of the os calcis, to the first bone of the little toe; the middle of another slip should then be placed under the bottom of the os calcis, and its ends extended perpendicularly up on each side of the leg; the third is to be applied along the foot, parallel to the first, and overlapping the half of it; the fourth should be placed parallel to the second, overlapping the half of it, and extending perpendicularly up the sides of the leg. In this manner they should be applied alternately along the foot and up the leg, the one holding and as it were antagonising the other in the motions of the foot, until the whole limb is covered from the toes to the knee. Subsequently to this, a calico bandage is applied in the usual manner, first alternately around the foot and ankle, and then up the leg as high as the knee. It is necessary to be particularly careful that the plasters and bandage be applied in such a manner that their superior and inferior edges are accurately placed in apposition to the skin, otherwise they will exert an unequal pressure, which is highly injurious. The whole should be applied with only that degree of tightness which is perfectly agreeable to the feelings of the patient, and not with a view of compressing the parts into a smaller space. In this manner every vessel in the limb will be uniformly and effectually supported." (P. 13.)

The renewal of the applications will depend upon the quantity of the discharge; for, when thus applied, they will remain for months without altering their position. By adopting this mode of treatment, an ulcer on the lower extremity is said to be placed under the same circumstances in respect to the circulation, as one that has its seat on the trunk, or on the upper extremity; and will heal with equal facility. Several cases are detailed to illustrate the efficacy of this practice.

Mr. Scott has found, from ample experience, that mechanical support may be employed with equal advantage in chronic inflammations of the upper extremity.

The author has seen many cases of chronic inflammation of different parts, in which the internal exhibition of mercury has been productive of great temporary benefit; but the already debilitated powers of the constitution are by this treatment so much impaired as to produce a subsequent aggravation of the disease. The local application of mercury, however, Mr. Scott assures us, has the same power of subduing chronic inflammation as when internally administered, and without producing its constitutional effect. This is a question of fact, which can be determined only by experience, and cases are related to show the grounds upon which the opinion rests. In many cases, mechanical support alone will be found capable of arresting chronic inflammation. In others, it may favor and expedite the subsidence of disease which it has not the power to remove. In these latter instances, mercury locally applied is capable of controlling the diseased action as effectually as it arrests acute inflammation when internally administered. Mechanical support, too, by relieving vascular distention, favors the operation of the above remedy as effectually as unloading the vessels by bleeding. When the situation of the part, then, renders it practicable, the two agents are combined.

Constitutional disorder is at the same time to be attended to. This practice equally applies whether inflammation has proceeded to ulceration or not. The majority of scrofulous ulcers, also, the author assures us, will be successfully treated by the same means. Common ulcers, whether varicose or not, when of very long standing, require more forcible compression. The first application of the plaster may be found to give some pain, but after a short time it will subside. If, however, the whole limb were encircled with this degree of tightness, the venous circulation would be impeded, and the inflammation aggravated. To stimulate the vessels of the affected part by a suitable degree of pressure, the plasters must only extend to half the circumference of the leg, and a short distance both above and below the seat of the inflammation. The application of lunar caustic will sometimes be found an useful adjuvant to the plasters.

*Diseases of the Joints.*—It is well known that, however similar these diseases become in their latter stages, they originate in different structures. From the appearance presented at an advanced stage, it is often impossible to distinguish which structure was primarily affected. This want of distinction Mr. Scott considers of less practical moment than might have been supposed, as the disease, although modified by the structure in which it is seated, essentially consists in chronic inflammation and its consequences. If disease does begin in one particular part of an articulation, the different textures soon participate in the disease, on account of their intimate connexion. The knee and hip joints are more frequently the seats of disease than the other articulations. Mr. Scott has therefore selected them for the subject of his remarks. The same principles and treatment will apply to the diseases of the smaller joints.

Passing over the description of the rise and progress of diseases of the joints, which is in substance similar to that of other writers, we arrive at their treatment. The constitutional means recommended by Mr. Scott are such as have already been pointed out by Mr. Brodie and other authorities. Upon the subject of general bleeding, Mr. S. perhaps goes a step further than his predecessors; for he says that, "even in the most acute stages, and most inflammatory forms of the diseases of the joints, there is usually little occasion for general depletion."

The author now proceeds to the especial object of his work, to explain the local treatment which, in the practice of his father and himself, has been very successful in these formidable ailments. If our limits permitted, we would willingly abstract the whole section devoted to this part of the subject, as it is for his local management in particular that Mr. Scott has gained so much celebrity.

If exercise is painful, either at the moment or shortly after, the patient must be confined altogether to the horizontal position. A fresh accession of inflammation is often produced by an accidental slip. Local bleedings must be had recourse to, to an extent proportioned to the severity of the inflammation. The object, however, is merely to produce a local effect, and not to influence the constitution. Cupping will be found very eligible in hip cases, but Mr. S. has sometimes thought that the pressure of the glasses has aggravated the disease when applied to the knee. In patients much emaciated, local bleeding has rather increased than reduced the disease; yet subsequently, in these very patients, when their general power was improved by proper means, local bleeding has been distinctly serviceable. Warm poultices are to be applied after a sufficient quantity of blood has been removed. Mr. Scott objects to cold applications By these means the disease will be reduced to a chronic state, and further bleeding will not be required unless any increase of inflammation should occur.

The active stage of the disease having been thus subdued, counter-irritation must be employed; but we are not to produce by it any disturbance of the constitution. The purpose of counter-irritation will be frustrated if the system generally is excited; for the rapidity of the circulation throughout the whole body, and consequently through the diseased limb, will be increased. The degree of counter-irritation must, of course, vary according to the susceptibility of the patient.

Blisters are not thought to be well adapted to these diseases. Our own experience corroborates the following remark: "If they (blisters) are applied sufficiently near the seat of the disease to exert any influence over it, the inflammation they excite is frequently propagated to the morbid structure."

Caustic issues are not recommended. Of the use of moxa, Mr. Scott cannot speak from his own experience. He has seen it fail in the hands of others.

"The above-mentioned irritating, and sometimes very mischievous, remedies may be all superseded by the following treatment. In the first place, the surface of the joint, suppose the knee, is to be carefully cleansed by a sponge, soft brown soap, and warm water, and then thoroughly dried; next, this surface is to be rubbed by a sponge soaked in camphorated spirit of wine, and this is continued a minute or two, until it begins to feel warm, smarts somewhat, and looks red. It is now covered with a soft cerate made with equal parts of the ceratum saponis and the unguentum hydrargyri fortius cum camphorâ. This is thickly spread on large square pieces of lint, and applied entirely around the joint, extending for at least six inches, above and below the point at which the condyles of the femur are opposed to the head of the tibia; over this, to the same extent, the limb is to be uniformly supported by strips of calico, spread with the emplastrum plumbi of the London Pharmacopœia. These strips are about one inch and a half broad, and vary in length; some are fifteen inches, others a foot, others half these two lengths, and the shorter or longer are selected according to the size of the part round which they are to be applied. This is the only difficult part of the process. This adhesive bandage ought to be so applied as to preclude the motion of the joint, prevent the feeble coats of the blood-vessels from being distended by the gravitation of their contents in the erect posture, and thereby promote their contraction. Over this adhesive bandage, thus applied, comes an additional covering of emplastrum saponis, spread on thick leather, and cut into four broad pieces, one for the front, the other for the back, the two others for the sides of the joint. Lastly, the whole is secured by means of a calico bandage, which is put on very gently, and rather for the purpose of securing the plaster, and giving greater thickness and security to the whole, than for the purpose of compressing the joint. This is an important point; as otherwise an application, which almost invariably affords security and ease, may occasion pain, with all its attendant mischief." (P. 133.)

In some cases, where the skin is thick and indolent, irritation may be promoted by rubbing on a small quantity of tartar emetic ointment, before the application of the cerate. If it is desirable to prevent all motion of the limb, pasteboard splints may be applied externally to the plasters. A piece of pasteboard should be softened by soaking in water, and cut into the length, breadth, and form of splints. This kind of splint is infinitely preferable to those made of wood. They support the limb very effectually, and are more agreeable to the patient.

"The remedies thus applied will not require very frequent removal. The time during which they may be left undisturbed will depend chiefly on the necessity for a repetition of the bleeding, in which we must be guided by the degree of pain, or, when there are open abscesses, by the quantity of the discharge. Should neither of these influence the question, the only necessity for removing the dressings will arise from their having ceased to keep up any irritation in the skin. In some cases it will be necessary to reapply them every week: in the generality of instances they may be allowed to remain a fortnight, and in others for a longer time. Even where there are open wounds, I allow them to remain several days, or a week, being firmly convinced by experience that the presence of the matter does less harm than the frequent disturbance of the part. A strumous ulcer can scarcely be disturbed too seldom: nothing does so much harm as officious dressing and probing." (P. 138.)

The plaster bandage ought to be applied in such a way as to afford ease and comfort to the patient. If it occasions pain, either on its first application or subsequently, it is either applied badly or the part is not in a fit state for it.

"When the disease commences in the bones, as long as it is confined altogether to them, I do not think that the loss of blood is attended with so much benefit as it is capable of affording after the soft parts have become affected. In this form of disease, the local use of mercury is most especially beneficial; it should, therefore, be applied without delay, and its operation favored by exciting a considerable cutaneous irritation. It is necessary also, in this case, to prevent all movement of the joint, in order that the bone, in its softened state, may not be injured by the contusion it would experience in the exercise of the limb." (P. 146.)

When the synovial membrane is the seat of the more acute form of disease, the loss of blood will be more frequently necessary; the mechanical support and counter-irritation must be moderate.

It is an important question to determine whether, when matter is formed in the cavity of a joint, it is desirable to evacuate it or not? Mr. Scott's experience leads him to determine "that to make an opening into the cavity of a joint is, in almost all cases, an injurious mode of practice." He would trust to the application of the foregoing remedies, which he believes are as well adapted to the suppurative as to any stage of the disease.

The practice of squeezing the matter out of an abscess, when opened in any way, is considered improper. If the matter be allowed to escape without any interference, and the parietes of the abscess be moderately supported by a bandage, they will gradually contract and diminish the cavity, at the same time that its contents are as gradually expelled.

A detail of many very interesting cases of disease of the articulations, successfully treated upon the plan advised by the author, concludes the work.

The mode in which Mr. Scott produces counter-irritation upon the diseased joint appears to be the principal peculiarity which marks his practice. We have frequently had occasion to lament the general disturbance produced by the common methods of counter-irritation, whether effected by the repeated application of blisters, the use of caustic issues, or the application of moxa; and, if the plan suggested should be found to act efficiently upon the part without disturbing the general system, we certainly are indebted to Mr. Scott for a very important improvement in the treatment of the diseases to which he refers. It must be remembered, that the advantages of supporting the joint in the manner pointed out by Mr. Scott depend very much upon the *dexterous* application of the various plasters and bandages which he recommends.

*The Morbid Anatomy of the Brain.* By ALEXANDER MONRO, M.D. Honorary Member of the Royal Physical, and Member of the Medico-Chirurgical Societies of Edinburgh; Professor of Anatomy and Surgery in the University of Edinburgh, and President of the Royal College of Physicians; F.R.S. & F.A.S.E. &c. &c. &c. Vol. I. *Hydrocephalus.* Illustrated by Copper-plates.—8vo. pp. 200. Maclachlan and Stewart, Edinburgh, 1827.

THE author of this volume has for twenty-five years directed his particular attention to the organic disorders of the brain. He has already published, in DUNCAN'S Annals of Medicine for 1803, the results of his researches into the structure of the skull and brain, in that species of hydrocephalus in which the head is much enlarged. His further views upon the subject have been postponed until the present moment, from an anxiety not to state any opinions without mature reflection and ample observation.

Dr. MONRO has divided his subject into two parts, each of which is to be illustrated by engravings. The object of the first is to give a graphic and concise sketch of the disease called Hydrocephalus, or Hydrencephalus, a disease which is at once very prevalent and fatal.* The second volume will be dedicated to the history of Apoplexy, Epilepsy, and Mania, and to the effect of Injuries of the Head.

The first chapter includes some general remarks on the effusion of a fluid within the head,—on the physical and chemical nature of that fluid,—on its different seats,—and on the concomitant organic derangement. According to some authors, there is an accumulation of a small quantity of fluid within the membranes of the brain, during life and health, the pressure of which is supposed to be essential to the healthy functions of the brain. This opinion is said to be untenable, as it rests neither upon incontrovertible evidence nor upon analogy. The Messrs. WENZEL have reported that they found a small quantity of water within the inferior cornua of the lateral ventricles of the brain, in two of three criminals who had been guillotined. Dr. Monro observes, that "a small quantity of fluid is generally found within the ventricles of the human brain after the lapse of twelve or sixteen hours after death; but this is no proof of the existence of water in that situation during life, the fluid

* "According to Dr. COINDET, 20,000 die annually in France from this disorder. According to Dr. ALISON, 40 out of 120 patients died of hydrocephalus at the New-Town Dispensary of this city. And, from the Annals of the Universal Dispensary of London, published by Dr. DAVIS, 8 out of 45 died from hydrocephalus."

being generated by the condensation of the halitus, which may be observed to escape when the brain has been exposed, while it is still warm." (P. 5.)

Upon this point we may observe, that MAGENDIE has ascertained that, in living animals, a quantity of serous exudation always exists between the pia mater and tunica arachnoides, which passes readily from the surface of the brain to that of the chord.* Three different kinds of encysted tumors, containing water, have been found within the human brain.

"To the first the name *hydatid* has been given, from the watery nature of the contents of the cyst. The size of these hydatids varies from that of a millet-seed to that of an orange. They are not peculiar to the human body, but are more frequently found within the brain of the inferior animals. In my book on the Morbid Anatomy of the Gullet, &c. I have described several kinds of these. I met with an example in which a hydatid was lodged within the substance of the human brain. The patient, a stout man, twenty years of age, complained of constant headache, chiefly on the right side, followed by a dilatation of the pupil, and epileptic fits, which proved fatal. On dissection, the cranium was found to be much thinner on the right than on the left side, particularly the right parietal bone, which in many places *was not thicker than a wafer*. On opening the right ventricle of the brain, a cyst, about the size of a goose's egg, was found within it, filled with a watery liquor, and surrounded by a gelatinous matter, which did not adhere to the membrane lining the ventricle." (P. 9.)

One of the most extraordinary circumstances connected with the history of hydatids, which Dr. Munro believes he discovered, is, that those parts which are in the vicinity of the animal are wasted: thus the skull is rendered soft, and thus hydatids pass out from the organs within which they have been originally imbedded, as from the liver or ovarium, into the cavity of the abdomen; and, from a similar cause, they are thus occasionally discharged from the body by the anus or skin.†

Preternatural encysted dropsical tumors are not unfrequent in the choroid plexuses. A cyst containing a yellow-coloured serum is often found within the substance of the brain of those who have fallen victims to apoplexy, and it has been said that their number corresponds with the number of apoplectic attacks. "Cysts of this description originally contain blood, the red globules of which are removed by absorption."

* Journ. de Phys. Exper. vol. v. p. 36.

† See MUNRO's Morbid Anatomy of the Gullet, Stomach, and Intestines.

In hydrocephalus, the watery fluid—

"generally occupies the four large ventricles of the brain, as these freely communicate with each other, and the quantity of the fluid depends generally upon the duration of the disease, and varies from an ounce or two to several pounds. When a small quantity of fluid is lodged within the ventricles, these undergo no perceptible change as to size or form; but, when several ounces of fluid fill them, they become considerably and externally enlarged, and partially distorted in form; and the still further accumulation of a fluid gives occasion to the disjunction of the bones of the skull.

"As the third ventricle is enclosed within the thalami nervorum opticorum, it is not so much enlarged as the lateral ventricles. The enlargement of the communication between the lateral ventricles keeps pace with that of the other constituent parts, and it has sometimes attained so large a size as to give passage to the little finger." (P. 13.)

The enormous size the head sometimes attains when there is a considerable quantity of fluid accumulated, is well known. Dr. M. met with one instance of a child that died when sixteen weeks old, whose head measured, at its greatest circumference, twenty-four inches. In chronic hydrocephalus, the brain resembles a bag filled with water.

"When the accumulation of fluid within the head amounts to several pounds, the brain resembles a bladder filled with water, the greater number of the convolutions being effaced. Upon examining the parietes of this bag with attention, they are found to be various in point of thickness, in different instances, and also in the same case in different places; and in some there is no vestige of the brain to be discovered on that side on which the patient used to lie in bed; and the internal surface of the enlarged ventricles is white." (P. 30.)

Dr. Munro has seen the pineal gland converted into a bag which was filled with water. In hydrocephalus, the lymphatic glands at the back of the head and upper part of the neck are very frequently enlarged, and somewhat indurated.

It appears, from the dissections of animals made by the author, that the brain of the most healthy is sometimes softer than usual. The result of his investigations as to the consistence of the brain of those who have died from hydrocephalus is, that *generally* it is neither harder nor softer than the healthy state. Dr. Monro observes, that the softening of the brain has been imputed to chronic inflammation, but it is perhaps rather the consequence of debility.

"The effect of low diet and debilitating diseases in inducing a softening of the brain, is familiar to those who have devoted much attention to anatomy; and the remarkable softness of the brain of criminals, which I have frequently noticed, may perhaps be imputed to these unfortunate persons being kept on very low diet for some time prior to their execution." (P. 37.)

The enlargement of the ventricles of the brain is a striking feature in many instances of hydrocephalus, and it has been explained in two different ways. Some authors have affirmed that it is owing to an extension of the ventricles, and others to the abstraction of a part of the substance of the brain.

"When the enlargement of the ventricles is not accompanied by the disjunction of the bones of the skull, unless it be supposed that the substance of the brain is compressed into less bulk, a part of it must be removed; and, if it be not removed, instead of being softer (as sometimes happens) the brain in that case should be invariably found to be reduced to a much firmer state, as may be done artificially, under the exhausted receiver of an air-pump.

"This abstraction of a part of the brain takes place, not only in hydrocephalus, but also when abscesses, cysts, or tumors of different descriptions, are formed within the substance of the brain. In order to make room for such abscesses or cysts, a part of the solid substance of the brain must be absorbed.

"As an argument in favor of the abstraction of a part of the brain, it may be added that, in the Museum, there is the head of a calf which contained fifteen pounds of water, while the brain weighed eleven drachms only.

"On the other hand, when the component bones of the skull give way, the ventricles of the brain may be much enlarged by extension; and if there be no vestige of brain on the side of the head on which the child has lain, and which I state on the high authority of Sir E. Home, that circumstance could only have taken place by absorption." (P. 38.)

After having noticed the various seats of the effused fluid, and the consistence of the brain when water is contained within the ventricles, Dr. Munro proceeds to describe the organic derangements of the membranes of the brain, and the nature of those tumors which are occasionally found attached to the surface, or are imbedded within the substance of the brain, in cases of hydrocephalus.

*Of the different kinds of Hydrocephalus, their symptoms, prognosis, and method of treatment.*—The first section of this chapter treats of hydrocephalus, in which the skull retains its natural size and form. Of this form of the disease, there are two very different varieties.

"The one is decided in its character at the outset, very rapid in its progress, proving fatal in three, four, or five days, is very rare, and has not been described by any author with whose works I am acquainted;—the other is frequently obscure in its origin, is much slower in its progress, being generally of three, four, or five weeks' duration; is very frequent, and has already been described by many at great length." (P. 69.)

*Of the most acute species of Hydrocephalus.*—This rare form of the disease is very sudden in its attack, and there are no previous symptoms denoting a derangement in the functions of the nervous system.

"It begins like the croup. The child awakes in the night in a state of extreme agitation, and much flushed, and with a quick pulse; he is hoarse, and the sound of the voice when he inspires is similar to that in croup: the sound seems to come from a brazen tube, which is contracted at a certain part.

"Children who are stout and healthy are equally liable to this disorder as the feeble and emaciated. And I have seen a patient, on the very day he was attacked by this disorder, who seemed very cheerful, and took his meals well, and was to all appearance in perfect health.

"The giving an emetic relieves the breathing, and, upon examining what has been rejected by vomiting, it is found to be evidently undigested." (P. 70.)

In illustration of the nature of this "very acute and fatal kind of hydrocephalus," three cases are given; two of which occurred in infants, the other in a woman sixty-five years of age. Dr. Munro was informed by Professor Burns, while his work was going through the press, that he had described, in his "Principles of Midwifery," this form of the disease. He attributes it to an affection of the origin of the eighth pair of nerves, induced by the state of the extremity of the fifth in dentition acting on its origin, which is near the eighth. Mr. Burns observes, that "it by no means necessarily ends in hydrocephalus, but it sometimes does, as, after the child has been apparently weak for weeks or months, he is carried off by hydrocephalus; which change is first indicated by general convulsions. Few children recover when the original attack is accompanied with convulsions, yet the case is not altogether hopeless. The symptoms enumerated by Dr. Munro as constituting this "most acute form of hydrocephalus" must be immediately recognised by every practitioner of moderate experience in infantile diseases. But we altogether dissent from the application of the term hydrocephalus: it is a positive misnomer. We grant that effusion within the head may occur at some uncertain period after this attack, but we take upon ourselves to assert that such an event very rarely follows. The effusion of water within the head, when it does occur after these symptoms, is a contingent and an unusual event, dependent generally upon some well-marked predisposition to cerebral affection, and certainly can have no claim to the rank of an essential disease. We must be allowed to speak with confidence, for we have had much experience upon the subject. It is this misapplication of the term "hydrocephalus" that leads to "heroic" doses of calomel and large bleedings, when, in the majority of the cases to which we are referring, purgatives and a free division of the gums, if the attack appears to be connected with painful dentition, will be found sufficient to relieve the symptoms. The spasmodic respiration, and croupy noise in breathing, sometimes continue for months.

*Of Acute Hydrocephalus.*—Dr. Munro states that this form of the disease "is most frequent in persons of a scrofulous constitution, where there is little energy of the system, and little activity in the vascular system." From the result of our own experience, we should say that this disease takes place most frequently in robust and vigorous children, but that it occasionally attacks those of debilitated constitutions. Dr. Munro has seen several instances in which a considerable quantity of water had been collected within the ventricles of the brain, and in which these ventricles had been considerably enlarged; notwithstanding which, there were no peculiar symptoms which indicated its presence. The disease sometimes begins like acute rheumatism, and the symptoms of disease in the brain do not show themselves until three or four days before death.

Dr. MONRO, senior, has observed another peculiarity, which has frequently attracted our own attention. "In some cases of hydrocephalus internus, in which the retina seemed to have very little sensibility, I have seen the pupil remarkably dilated when the eye was exposed to a bright light; and, when the light was removed, it was sensibly lessened."

There are instances in which the disease has terminated favorably after convulsions, blindness, and delirium had taken place, and when the patient had been supposed to be dying; and, what is very remarkable, even the sight has been regained.

*Of the Cause of the Effusion of Water.*—A morbid accumulation of blood in the vessels of the brain, sometimes proceeding to a degree of inflammation, and generally, but not always, producing an extravasation of a watery fluid, is considered by most authors to be the originating cause of this disease.

Dr. Monro has brought forward a variety of arguments for the purpose of showing that this disease is generally unconnected with inflammatory action of the brain. We apprehend the fact may be briefly stated. Like every other form of dropsical affection, that which occurs within the cranium may be dependent upon debilitating causes, but, in the majority of cases, the symptoms of this disease, and the constitution of the child, clearly indicate a high state of cerebral excitement, if not the existence of positive inflammation of the brain. Dr. Monro observes, that

"Some advocates for the opinion that hydrocephalus originates from inflammation of the brain, have imputed the disorder to a fault in the digestive organs. But a fault of the digestive organs, so far from adding to the vigor of the constitution, produces a very contrary effect, and, by diminishing the vis vitæ, tends to avert or to remove a disposition to inflammation." (P. 106.)

Now this argument is more plausible than solid. We grant that derangement of the digestive organs will diminish the general powers of the constitution; but nothing is more common than to detect one organ labouring under great accumulation of blood, and even inflammatory action, while the system generally is in a state of weakness. Local bleedings are frequently necessary, when general support is not contra-indicated.

We should be strongly inclined to doubt the experience of any practitioner in the diseases of children, who, from any abstract notions, should deny that derangement of the digestive organs very frequently produces a high and dangerous degree of cerebral irritation, and even inflammation.

It is objected by the author, that, "if this disease originates from inflammation, mercury is a very improper remedy. Mercury is a stimulant, not a sedative." To this doctrine we reply, that mercury is not, or ought not to be, employed until the general excitement produced by inflammatory action has been reduced by bleeding and other proper means; and that then we have nothing to fear from the judicious use of it. We should remember, also, that mercury acts more on the extremities than on the centre of the vascular system, and that, during inflammation, the capillary extremities are in a state of debility, and require the excitement which mercury appears capable of imparting to them. For many excellent remarks upon the subject of the employment of mercury during the existence of inflammation, we refer our readers to Lucas "on the Principles of Inflammation and Fever," a work which will amply repay the trouble of an attentive perusal.

*Prognosis.*—The prognosis of this disease is generally unfavorable, but every practitioner must "have met with instances in which all the more usual characteristic symptoms of the effusion of water were present," and still, by the use of proper remedies, the patients have recovered.

*Of the Treatment of Acute Hydrocephalus.*—To remove any derangement of the functions of the alimentary canal, calomel and other purgatives are recommended. Torpor of the intestines is said to be a consecutive symptom: it merits peculiar notice, as indicating derangement in the functions of the brain, "in which case a large blister should be applied over the head." We have, however, frequently seen the cerebral disturbance much increased by the application of blisters to the scalp. Calomel, combined with James's powder, is the favorite prescription of Dr. Monro, to restore the healthy functions of the bowels. Dr. Cheyne also thinks very highly of James's powder in this disease. The diet of the patient is to be strictly attended to.

"If the disease be supposed to originate in debility, in laxity of the brain and its vessels, it may possibly be averted, by avoiding cold, all vicissitudes of the weather, and every means by which the bodily strength may be impaired; and by endeavouring to improve and invigorate the constitution, by generous diet, wine, and the keeping the bowels regular; and by removing irritation, and the irregular action of the chylopoietic viscera, by warm clothing, and moderate and daily exercise in a pure atmosphere.

"By adopting such a mode of treatment, I think I have had the satisfaction of averting the disease.

"But, on the other hand, should the disease be supposed to proceed from the over-excitement of the vessels of the brain, and if inflammation be the primary cause, and the effusion merely the effect, an attempt should be made to remove that state by the general and topical detraction of blood, low diet, by refrigerant applications to the head, by purgatives, and by setons applied to the neck, by spices and blisters, and by avoiding all such causes as induce plethora." (P. 127.)

In our opinion, the latter plan of treatment will be indicated in most cases.

The next section treats of *Hydrocephalus accompanied by an Enlargement of the Skull."*—Mercurial frictions upon the head have frequently been of use in this form of the disease. Dr. Traill communicated an interesting case of this kind to the author. The late Mr. Wilson has also mentioned the effect of friction with mercurial ointment in diminishing the volume of the skull. He says,

"I may be permitted to observe that, when the cause is removed, the bones will cease to enlarge; and in some instances, when the increase has not been carried too far, will recover their natural structure. I think it right to add, that in one family, where two children had died of water in the ventricles of the brain, four others were in succession attacked with the strongest-marked symptoms of the disease, attended with much enlargement of the head, who, by attention to the state of the gums, and mercurial frictions to the head and back, are now alive, well, and with as much mental intelligence as other children of the same years." (P. 144.)

Bandages to the head have occasionally been serviceable; but, by their pressure, convulsions have sometimes been caused.

"Considering that the symptoms of oppressed brain are relieved by the disunion of the bones, I should suppose that the pressure of a bandage put around the head would generally produce the same effects as Mr. Hood has described; and hence it is only of use after the water has been artificially drawn off, in preventing the deliquium which will generally succeed the puncture." (P. 145.)

As other remedies are usually of no avail, it has been recommended by SEVERINUS and LE CAT to draw off the water by puncturing the brain. Dr. Monro has no confidence in this practice. It appears, however, to have sometimes been followed by temporary relief. Sir E. Home, Dr. TRAILL, and Mr. BROWN,* have related cases in which water was repeatedly drawn off by puncture, and always with relief, "and temporary retention of sight and faculty of attention, but it ultimately ended fatally."

The concluding chapter of the work contains some observations upon the effect of pressure, tumors, &c. upon the brain.

In an Appendix, a communication is given from Dr. KELLIE, upon tubercles, and on the effects produced by these formations on the different textures and organs in which they are found.

Although upon some points we have dissented from the opinions of Dr. Monro, we may, without any critical inconsistency, recommend his work, as a whole, to the attention of our readers. It contains much useful information.

---

*Transactions of the Association of the Fellows and Licentiates of the King and Queen's College of Physicians in Ireland.* Vol. V. —8vo. pp. 576. J. Cumming, Dublin; Longman and Co. London, 1828.

(Continued from p. 525.)

WE resume our analysis of the Papers, properly so called, which are contained in this volume; having placed the most interesting of the detached cases in another department of our Journal.

The next paper, in the regular order of succession, is one on the *Indications afforded by the sensible Qualities of Plants with respect to their Medical Properties.*" It is from the pen

* London Medical and Physical Journal, vol. xii. p. 102.

of Dr. Osborne, and, though sufficiently interesting, is not strictly of a practical nature, and indeed contains but little matter of novelty. We therefore pass on to *Cases intended to illustrate the Application and Utility of the Stethoscope,* by Dr. Townsend. The author remarks, that the importance and utility of mediate auscultation are not yet recognised sufficiently to produce its general adoption, and he has therefore detailed some cases in order to prove that auscultation is capable of affording an "extraordinary" precision in distinguishing the most complicated forms of pulmonary disease.

The first case adduced is one of pleurisy and pneumothorax, with a fistulous opening between the bronchi and pleura. The following are the details:

"On the 25th of March, 1827, in compliance with Dr. Cheyne's kind invitation, I visited John Munro at the Royal Infirmary, a tall, well-proportioned dragoon, thirty years of age, of whose previous history I learned the following particulars: His complaints commenced in October last, with cough, pain in the chest, and diarrhœa, for which he was bled, blistered, &c. A recurrence of the same symptoms called for a repetition of these measures, which, as well as several others since employed, failed to produce any permanent advantage.

"At the time of my visit he was up and dressed, walked about the room, but was soon out of breath, and easily fatigued. He was considerably emaciated, had much dyspnœa, not sufficient, however, to materially affect his speaking; profuse night-sweats, diarrhœa, thirst, anorexia. Pulse 120, small and vibratory; number of respirations, thirty. Cough most troublesome on awaking in the morning. Sputa apparently mucous, are stated to have diminished considerably in quantity within the last three weeks, from which period is also dated the aggravation of his dyspnœa. On viewing the thorax, the right side appeared considerably more dilated than the left, especially anteriorly and laterally, at its lower half. Percussion employed over the dilated surface elicited a clear hollow sound. In this space, too, the respiratory murmur was perfectly inaudible; but, immediately after coughing, a peculiar sound, resembling the vibrations of a porcelain jar when gently struck, (*tintement metallique,*) was distinctly heard in a space corresponding to the posterior convexities of the sixth, seventh, and eighth ribs. This sound was not produced either by inspiration or speaking.

"Succussion did not produce the sound of fluctuation, although the patient said he felt water dashing against his side. In the superior part of the same side of the chest (the right) the dilatation was scarcely, if at all, perceptible. The sound, on percussion, not particularly sonorous, and the respiratory murmur audible posteriorly.

"At the left side, the sound on percussion was natural, though considerably duller than at the right. Respiration was distinctly audible all over the lung's surface, except in the space corresponding to the superior lobe, where cavernous respiration and cough, with perfect pectoriloquy, were heard distinctly.

"*Diagnosis.*—A tubercular cavity occupies the upper lobe of the left lung.

"The dilatation of the right side of the chest is produced by pneumothorax, and the co-existence of the *tintement metallique* proves that the air in the pleura proceeds from a communication between the bronchia and pleura. The medium of communication in this case I conceive to be a tubercular cavity; first, because such is by far the most frequent mode of communication between those parts; and, secondly, because the existence of a tubercular cavity in the opposite lung converts the probability of this species of abscess into a moral certainty, of which no doubt could have existed, if the patient had been examined with the stethoscope, and pectoriloquy found under the right clavicle, before the accession of the pneumothorax.

"I attribute the comparatively dull sound, on percussion, of the superior part of the thorax, and its less degree of dilatation, to the existence of ancient adhesions, which prevent the air accumulating in that region, between the pleura costalis and pulmonalis.

"To recapitulate: The lesions I expect to find are, a tubercular cavity in the upper lobe of the left lung; the right side of the thorax distended with air and fluid, (the latter at present exists in small quantity, but its proportion will no doubt go on increasing;) in the right lung a tubercular cavity, communicating with the sac of the pleura on the one hand, and with the bronchia on the other, allowing the air inspired to pass freely into the pleura; and, finally, the superior lobe united by old adhesions to its corresponding costal pleura.

"This detailed diagnosis was written and handed over to Dr. Cheyne on the evening of my first visit. * * *

"*Dissection, forty hours after death.* Present, Drs. Cheyne and Stack.—External appearances: Body well proportioned; considerable emaciation; legs and feet slightly œdematous; the right side appeared considerably more dilated than the left, but, on measuring with a tape, the greatest difference was found not to exceed one inch and a half.

"On employing succussion, fluctuation was heard by applying the ear to the chest, but was not audible to the bystanders.

"The head was not examined.

"Thorax, (the right side:) A trocar was introduced between the fifth and sixth ribs, near their junction with their cartilages: an immediate rush of air followed, part of which I succeeded in collecting in a large phial filled with water, and inverted over the canula.

"On removing the sternum, a vast unoccupied space was observed in the anterior part of the thorax, capable of containing fully two quarts of water. This space had been occupied by air, which may consequently be estimated at that quantity. The lung just appeared above the surface of the fluid, which occupied the posterior region of the thorax: it was closely compressed against the spine, and seemed reduced to one-third of its natural dimensions. The fluid effused might be in quantity about two quarts, was of a yellowish green colour, tolerably clear at its surface, but rendered turbid at bottom by numerous fragments of opaque, puriform flocculi of albumen.

" Before touching the lung, in order to guard against an accidental formation of the opening, which I expected to find, an incision was made into the trachea, and the pipe of a pair of bellows introduced. The air passed freely through the lung, and appeared in bubbles at the surface of the fluid, in which it was immersed.

" The fluid being removed, the upper lobe of the lung was found in close contact with, and firmly attached to, the costal pleura.

" The whole surface of the lung, except where it was attached, was coated with an albuminous exudation of a dirty white colour, of several lines thickness, its surface wrinkled, not unlike the rind of a shrivelled apple. The costal, mediastinal, and diaphragmatic pleuræ, were still more thickly coated with this exudation, which, though firmly attached to the subjacent pleura, and apparently incorporated with it, might, by careful dissection, be separated from it, leaving the membrane underneath in a state of perfect integrity.

" The lung was now detached: on its anterior surface, about two inches from the summit of the upper lobe, was discovered a fistulous orifice, capable of receiving my little finger, its margin well defined, rounded, and nearly cartilaginous. A probe introduced passed readily through a series of small tubercular cavities into one of the principal bronchia. At intervals of half an inch below this fistulous orifice existed three small oval superficial ulcers, which, on close examination, did not appear to communicate with the bronchia. They were evidently formed by softened tubercles, developed immediately under the pleura; for, on different parts of the lung's surface, there were several similar oval nests of tubercles, some not yet softened, others quite soft, and elevating the pleura, through which they had not as yet formed a passage.

" Posteriorly near the root of the lung, and about the base of the superior lobe, immediately underneath its adhesion to the pleura, was another fistulous opening, of half an inch in diameter, which communicated by a long sinuous passage with a large tubercular abscess occupying nearly the whole upper lobe. This passage was lined all through by a highly vascular membrane, exactly similar to that which lined the tubercular abscess, having its surface coated with a layer of lymph. Into this vast abscess was also traced one of the principal bronchial divisions: its entry into the cavity was within a few lines of that of the sinuous passage above described.

"The middle and lower lobes contained several tubercles. The bronchial glands also were much enlarged, and studded with tubercles.

"The left side of chest.—This lung was studded throughout with granular tubercles of the size of duck-shot. In the superior lobe was found one cavity, capable of containing a large filbert, and communicating with two or three smaller ones. In the middle lobe, the tubercles were all opaque and whitish. In the inferior, many of them were in the first, or greyish, semi-transparent stage." (P. 138.)

In this case, the patient was able to sit up and dress himself till within a very short period of his death; whereas, Laennec states that, in all the cases he had seen, they were much oppressed and confined to bed. Nothing could be more accurate than the diagnosis in the preceding case: not so, however, that which follows.

"On the 7th of April, whilst walking through Dr. Ferguson's wards in the Whitworth Hospital, to which he has kindly allowed me admission, my attention was directed by the nurse to a patient, who, she stated, was labouring under the same disease as his predecessor in the same bed, who had died a few days before of diseased heart.

"The following is an outline of the case: Owen M'Kenna, æt. thirty-four, a flaxdresser. Thorax excessively deformed, the left side being quite flattened anteriorly. Two months ago was seized, after exposure to cold and wet, with shiverings, cough, dyspnœa, and violent palpitations on going up an ascent, or making any exertion. These symptoms increased progressively. At present his dyspnœa is extreme; violent palpitations; pulse rapid, full, and bounding; tremulous motion of jugulars, but no distinct regurgitation; inability to lie in the horizontal position; face of a dirty aguish complexion; lips and nails of a dark leaden hue; feet and ankles œdematous; cough soft, in long paroxysms; sputa occasionally tinged with blood. Appearance altogether highly indicative of morbus cordis. Says himself that the beating of his heart causes the bed to shake under him. As he appeared to suffer very much, I only applied the stethoscope to where I expected to find the seat of the disease.

"The heart was felt to pulsate vehemently in a space of three inches circumference, over the cartilages of the fifth, sixth, and seventh ribs. Pulsations stronger over the left than over the right ventricle. The rythm of the contractions appeared regular, but their sound somewhat duller than natural. Number of pulsations 120, of respirations 44. Heart's action so tumultuous as to cause the whole anterior surface of the chest to vibrate under the hand when applied.

"These physical signs so far confirmed the impression which the symptoms had previously made on my mind, that (not having the case to treat) I pursued my examination no further, but entered as my diagnosis, *Morbus cordis; hypertrophy of both ventricles, especially the left.*

"In a few days after I again saw the patient: dyspnœa rather increased; lips more livid. I wished to examine the state of the lungs, but he was unable to sit up, as giddiness immediately came on. Anteriorly a ronchus crepitans was heard all over the præcordial region, and all down the right side of the chest. I, in consequence, added to my former diagnosis, *double pneumonia.*

"The heart's action continued tumultuous, and in a few days death put an end to his sufferings.

"*Dissection, twenty hours after death.*—To my great surprise, the heart was found perfectly well proportioned,—at least no alteration of its structure was observed at all sufficient to account for the great derangement of its functions. For, although the opening of the foramen ovale was not perfectly closed, yet so effectually did the valve overlap and protect it, that no blood could pass from the one auricle to the other, unless the contracting force of the one far exceeds that of the other; but in this case both auricles maintained their due relative proportions, both as regarded their capacity and the strength of their parietes.

"The lungs did not collapse in the least, and adhered firmly to the costal pleura throughout its entire surface.

"The right lung felt perfectly hard, and, when cut, exhibited a surface studded with miliary tubercles, in their different stages of development, almost as close as they could be packed. Those points not occupied by tubercles were just passing into the stage of hepatization.

"The left lung contained in its upper lobe a cavity capable of containing an orange, and communicating freely with the bronchia. The root of the lung had quite an indurated feel, so crowded was it with tubercles. The pneumonia at this side had not passed its first stage (*engouement*). (P. 152.)

Dr. Townsend is of opinion that in this case the pulsations felt over the chest were produced by the consolidated lung transmitting the impulse of the heart. So far, however, from looking upon it as militating against the stethoscope, he adduces it as an argument in its favor: had he examined the superior lobe of the left lung under the clavicle, or in the supra-spinal fossa, he feels convinced that he would have detected the true nature of the case. One thing its relation undoubtedly proves—very great candour. That a case in which the diagnosis was so egregiously wrong, can be converted into a circumstance favorable to the stethoscope, is straining rather too far the courtesy of his readers.

The next case is intended to show the character and stethoscopic phenomena of hysteric cough, and the intimate connexion between precision in diagnosis and success in treatment.

"Mary Bohey, æt. forty-five. On the 25th of January she again applied at the Talbot Dispensary, stating that her strength returned but slowly, that her appetite was bad, and that her spirits were so low that she used frequently to burst into involuntary fits of crying. To these ailments was superadded, about a fortnight since, a cough, at first slight, but daily becoming more urgent, until at present it comes on in paroxysms of fifteen minutes long, during which the face becomes purple, the eyes suffused, as if starting from their sockets, the head feels ready to burst asunder, and the chest is violently constricted. In short, she seems in imminent danger of suffocation. These violent paroxysms are never succeeded by expectoration, and yield only when the patient is perfectly exhausted. The longest and most urgent fits come on when she first goes to bed, and when she gets up in the morning. Pulse 120, small and vibratory; bowels constipated; has occasional attacks of colicky pains, during which the abdominal muscles are spasmodically constricted.

"On account of her great fatness, no information could be derived from percussion.

"On applying the stethoscope, the respiratory murmur was heard quite pure all over the anterior surface of the thorax. Posteriorly at the right side, respiration pure. At the left side, respiratory murmur also pure, but much more feeble than at the right.

"This examination was made during an interval between the paroxysms. The agitation, however, excited in the patient's mind brought on a few convulsive sobs, which were quickly followed by a most frightful paroxysm of coughing, during which she literally gasped for breath.

"I again applied my ear, and was surprised to find the respiratory murmur perfectly inaudible all down the left side posteriorly, where but a few minutes before I had heard it distinctly, though more feebly than at the opposite side, where also the respiration was scarcely to be heard, except during the forced inspirations which immediately preceded the act of coughing.

"The symptoms of hysteria in this case were too evident to be mistaken, and the probability of the cough being only a sympathetic affection was, by the result of auscultation, converted into certainty. My treatment was directed accordingly, and I ordered ʒj. of the powdered Valerian to be taken three times a day; a practice which I had seen employed with eminent success at Naples. Three doses were taken on the first day: cough less troublesome on lying down; enjoyed some sound sleep during the night. After the fourth dose, the cough no more came on in paroxysms; other symptoms, too, experienced a proportionate improvement; and on the third day she was perfectly tranquil, and free from cough." (P. 158.)

The principal stethoscopic interest of this case, we are told, consists in the sudden cessation of the respiratory murmur, when it had been but a short time before distinctly audible; and when it again returned, after the paroxysm was over. This Dr. Townsend supposes may have depended upon spasm of the transverse fibres of the bronchial tubes: he adds, that these always acquire increased violence in every case in which protracted dyspnœa takes place. For further information respecting spasmodic affections, we are referred to LAENNEC's " immortal treatise."

The particulars of the next case we shall not detail, but content ourselves with stating generally that it was one of fever, in which, during the convalescence, the patient became affected with pneumonia, unattended by the usual characteristics; for the detection of which the Doctor was indebted to the stethoscope, and which he was thus enabled to treat in an efficient manner.

In an appendix to his paper, Dr. Townsend relates another case of pneumo-thorax combined with pleurisy.

" Wm. M'Nally, æt. twenty, was admitted into Dr. Orpen's ward in the Whitworth Hospital on the 24th of October, 1827. His general appearance is that of a robust man, labouring under acute inflammatory disease; his face flushed, skin hot, and pulse rapid (160); his breathing laborious and hurried (44). At each inspiration, the alæ nasi dilate widely; the left side of his chest heaves greatly, whilst the right continues nearly immoveable; the margin of the liver is felt below the ribs, but the hand laid on the abdominal muscles can detect no motion either in them or in the diaphragm of the right side; his chest is very much distended at the right, especially anteriorly and laterally; the difference of circumference between the sides (measured from the xiphoid cartilage to the spinous process of the opposite vertebra) amounts to an inch and a half. He lies exclusively on the dilated side; coughs a great deal, and in violent paroxysms; expectoration copious, and appears to consist wholly of mucus. Says that he feels no pain whatever, nor can he recollect any sensation of tearing, or of violent pain, suddenly affecting his side.

" On employing percussion, the dilated side yields a duller sound than the left: this, however, is evidently owing, in a considerable degree at least, to the infiltration of the subcutaneous cellular tissue of the right side, on which he constantly lies. Both anteriorly and posteriorly, the natural respiratory murmur is extinct all over the right side of the chest; and from the eighth rib upward its place is supplied by a loud *bourdonnement amphorique*, accompanied, when he coughs or speaks, by a well-defined *tintement metallique*. No fluctuation is heard on succussion, or change of posture. At the left side the respiration is puerile, and the bronchial tubes are felt vibrating under the hand when applied over the supra-spinal fossa.

" *Diagnosis.*—Pleuro-pneumo-thorax, with fistulous communication between the bronchia and the right sac of the pleura, is unequivocally demonstrated by the stethoscopic phenomena. The cause of this communication is not, however, so apparent; his appearançe, and the history of his previous health, seem incompatible with the development of tubercles to such an extent as is usually observed in those cases where their bursting into the sac of the pleura gives rise to the extravasation of air, an event of infinitely rare occurrence, unless in the last stage of phthisis." (P. 484.)

The symptoms gradually increased, the principal alteration in the stethoscopic phenomena being that fluctuation had become evident in succession, and that, when he sat up in bed, a drop was heard to fall with a peculiar metallic sound striking upon the surface of a fluid, about the level of the seventh rib; below this, the sound on percussion was dull. Suffocation being threatened, it was resolved to perform the operation of paracentesis thoracis.

" The patient being seated in bed, an incision was made by Mr. M'Dowell between the eighth and ninth ribs, (counting from above downwards,) at about three inches from the spine. When the muscles were divided, fluctuation was distinctly felt through the pleura: the division of this membrane was followed by a violent gush of yellow, puriform fluid, of a peculiarly heavy, disagreeable smell; at first this fluid came away in a continued stream, but its flow soon became interrupted, each jet corresponding to an effort of expiration. When about three pints, or rather more, of this fluid had flowed out, a loud rush of air followed, and continued for some minutes to issue from the wound, with considerable force, at each expiration.

" At this period of the operation, the patient uttered two or three agonising groans, and appeared on the point of fainting: the wound was immediately closed, and he quickly recovered his composure and the use of his voice, expressed himself greatly relieved, and his countenance brightened up considerably. The wound was dressed with adhesive plaster, and a flannel roller applied round the thorax. In half an hour after the operation, the pulse had improved considerably in strength and regularity, though its rapidity was scarcely diminished (150); his respiration, though much less laboured, retained its frequency (40). The alteration in the stethoscopic phenomena was very striking; the *tintement* and *bourdonnement* had totally disappeared, but the voice reverberated loudly, as in a vast phthisical cavern." (P. 490.)

The operation was performed on the 31st of October, and the patient, though he expressed much relief, gradually sank, and died early on the morning of the 2d November. About the middle of the lower lobe of the right lung was found a fistulous opening with indurated margins, capable of admitting the point of the finger.

*Sketch of a Medical Report on the Epidemic Dysentery which prevailed in Dublin in* 1825. By Dr. O'Brien.

Numerous well-marked cases of dysentery occurred at the period alluded to; but we shall confine ourselves to an account of the method of treatment found most successful. The patient, if young and robust, was bled to ten or fourteen ounces, provided he was seen in the first stage of the disease. He was then put into a warm bath at rather a high temperature. After this the whole body, but particularly the belly, was rubbed with camphorated oil; the patient was then swathed in a flannel bandage, drawn tightly round the abdomen, and put to bed. The next step consisted in the administration of a bolus, containing ten grains of calomel, one (or sometimes two) of opium, and occasionally combined with antimonial powder. This was repeated every eight hours, the anodyne effects of the opium not extending beyond that period. For the most part, the patients were brought to the hospital rather late in the day, and the measures above mentioned occupied the first evening and night after admission. Next morning, a purgative draught, containing castor oil, senna, rhubarb, and neutral salts, variously combined, but always having some tincture of opium, was exhibited. If the symptoms still continued urgent, the patient was now bled a second time, and the bath repeated, and the calomel and opium continued, alternated with purgatives, until the disease yielded, or the gums became affected. When this took place, the mercury was omitted, and the other medicines continued without it. In many instances, Dover's powder was substituted for opium, and this preparation is very highly spoken of by our author.

When patients were admitted who were more advanced in life, or when the disease had already passed its earlier stages, the bleeding was omitted, and the treatment with calomel and opium, alternated with purgatives, adopted. Here also the mercury was omitted as soon as the mouth was affected; after which the decoction of simarouba, with laudanum in sufficient quantity to mitigate the pain, was given every six or eight hours. With regard to the Simarouba, the author remarks, that " a tolerably extensive experience of its effects induces him to believe that it possesses considerable astringent powers, and that, combined with opium, it is an antispasmodic of no mean efficacy."

The next paper consists of *Clinical Observations made during the Epidemic Fever of* 1826, by Dr. Reid. The most interesting part of this communication consists in some cases in which the hydrochloruret of lime was exhibited, and which will be found among our Hospital Reports.

